# Microstructure and Chlorine Ion Corrosion Performance in Bronze Earring Relics

**DOI:** 10.3390/ma17081734

**Published:** 2024-04-10

**Authors:** Zhiqiang Song, Ojiyed Tegus

**Affiliations:** 1 Institute for the History of Science and Technology, Inner Mongolia Normal University, 81 Zhaowuda Road, Hohhot 010022, China; songzhiqiang@imnu.edu.cn; 2 College of Physics and Electronic Information, Inner Mongolia Normal University, 81 Zhaowuda Road, Hohhot 010022, China

**Keywords:** bronze earrings, alloy structure, NaCl solution, polarization curve, electrochemical corrosion

## Abstract

Chlorine ions play an important role in the corrosion of bronzeware. This study employs techniques such as XRD, OM, SEM, EBSD, and electrochemical testing to analyze the microstructure, crystal structure, chemical composition, and corrosion performance of bronze earrings unearthed at the Xindianzi site in Inner Mongolia. The results indicate the presence of work-hardened structures, including twinning and equiaxed crystals, on the earrings’ surface. With an increase in chloride ion concentration in NaCl solutions from 10^−3^ mol/L to 1 mol/L, the corrosion current density of the bronze earrings increased from 2.372 × 10^−7^ A/cm^2^ to 9.051 × 10^−7^ A/cm^2^, demonstrating that the alloy’s corrosion rate escalates with chloride ion concentration. A 3-day immersion test in 0.5% NaCl solution showed the formation of a passivation layer of metal oxides on the earrings’ surface. These findings underscore the significance of the impact chloride ions have on the corrosion of copper alloys, suggesting that activating the alloy’s reactive responses can accelerate the corrosion process and provide essential insights into the corrosion mechanisms of bronze artifacts in chloride-containing environments.

## 1. Introduction

Bronze, as one of the earliest alloys used in human history, played an irreplaceable role in the development of ancient civilizations due to its unique and exceptional chemical and physical properties [1]. The addition of tin and lead not only significantly improved the characteristic fluidity and formability of bronze, it established bronze as an indispensable material in modern industrial production [2,3]. China’s bronze civilization, with its unique artistic style and highly developed craftsmanship, demonstrated the prosperity and progress of this ancient society. In the central and southern regions of Inner Mongolia, archaeologists have discovered a significant number of bronze artifacts from the Eastern Zhou period, reflecting characteristics of the northern nomadic tribes and their cultural exchanges and integration during this period [4]. Notably, ancient bronze artifacts were primarily cast, however some were further processed through hot and cold working to enhance the alloy’s mechanical properties. Bronze earrings, in particular, often underwent intricate hot and cold processing, showcasing exquisite skills and craftsmanship of the ancient artisans’ and their pursuit of beauty. However, these precious bronze relics inevitably suffered corrosion after being buried underground for considerable lengths of time [5]. There are multiple metallic elements present in bronzeware, many of which can cause severe electrochemical corrosion and breed significant bronze diseases, leading to mineralization and even disintegration of the bronzeware. The rust on the surface of bronzeware can be divided into harmless rust and harmful rust. Harmful rust, also known as “powdery rust”, is predominantly composed of cuprous chloride and basic copper chloride. The chloride ions contained in it can cause corrosion, penetrating the surface and the inside of objects [6].

Bronze earrings, compared to other small copper items, have shown superior performance and integrity in resisting corrosion, sparking great interest among scholars.

The corrosion of ancient bronze artifacts has always been a focal point in archaeological materials science. Previous studies primarily focused on qualitative analysis of corrosion products [7,8,9,10,11,12]. Whereas these studies have provided valuable insights into the corrosion mechanisms of bronzes, the relationship between the corrosion behavior of bronze alloys and environmental factors, especially in specific geographic locations, climate types, and the combined effects of alloy composition, structure, phase distribution, and exposure time, requires further investigation [13]. The corrosion mechanisms of ancient bronze alloys are complex and diverse. These included preferential corrosion at impurities or non-pure grains, grain boundary corrosion, and pitting, amongst others. The impact of environmental conditions such as humidity, temperature, and anions on corrosion rate is also crucial [14,15,16,17]. Corrosion phenomena are closely related to the microenvironment of burial sites, particularly chloride ions, which are key factors in promoting the corrosion of bronze alloys [18]. Chloride ions in natural environments can promote the formation of copper oxides and chlorides on the surface of bronze alloys [19,20], leading to a corrosion layer known as patina, which not only is visually attractive but also acts as a stable protective layer [21]. Environmental changes can accelerate or decelerate the corrosion rate [22]. Studies in simulated buried and seawater environments have shown that the passivation layer of bronze alloys is related to the oxygen content in the environment [23]. Although the corrosion laws of bronze alloys have been widely studied [24]. However, the reason for the continued corrosion of the alloy after the excavation of bronze ware has always been a challenge. Some studies suggest that the study of the corrosion mechanism of bronze should be carried out on the surface covered with copper green, especially the CuCl layer [25]. Research has shown that CuCl, typically formed between the alloy and Cu_2_O layer, plays an important role in the chloride ion corrosion of copper alloys [26]. When corrosive chloride ions corrode alloys, Cu as the anode will be corroded into CuCl. It is an unstable intermediate because chloride ions are released during the hydrolysis and oxidation process of CuCl. However, research on the impact of chloride ion concentration and the effect of surface processing on corrosion performance remains relatively limited.

Based on the context provided, this study conducts a comprehensive analysis of bronze earrings unearthed from the Xindianzi cemetery in Inner Mongolia. Utilizing modern scientific methods such as XRD, OM, SEM, and EBSD, the research delves into the structure and chemical composition of the bronze earrings. Through electrochemical polarization curves and electrochemical impedance spectroscopy, the study investigates the corrosion behavior of surface-treated bronze earrings in various concentrations of NaCl solutions. The goal is to uncover a pattern and thus show how chloride ion concentration affects the corrosion of bronze alloys, providing significant reference data and insights into the study of corrosion performance of ancient bronzes.

## 2. Materials and Methods

### 2.1. Materials

**Figure 1:**
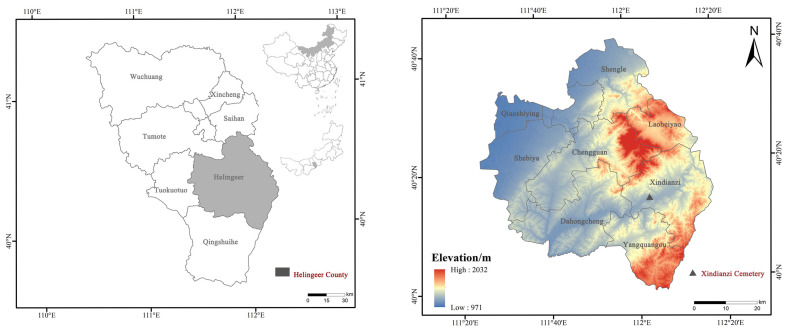
Schematic depiction of the location of the Xindianzi Cemetery during the Eastern Zhou Dynasty.

The M47 at Xindianzi Cemetery is a vertical pit tomb, with a length of 220 cm, a width of 80 cm, and a depth of 40 cm. The tomb owner is a North Asian male of around 45 years old. More than 1000 copper artifacts and 46 earrings have been unearthed from the Xindianzi Cemetery, and the alloy surface displays relatively stable corrosion. After excavation, the surface soil of the bronzeware was simply cleaned and then stored in the cultural relic storage room. The test samples originated from the Xindianzi cemetery in Inner Mongolia, from where the remains of a bronze earring from the Eastern Zhou Dynasty (Figure 1) M47 was unearthed. According to the cemetery briefing, it can be inferred that the Xindianzi cemetery dates back to the late Spring and Autumn period to the early Warring States period [27].

**Figure 2:**
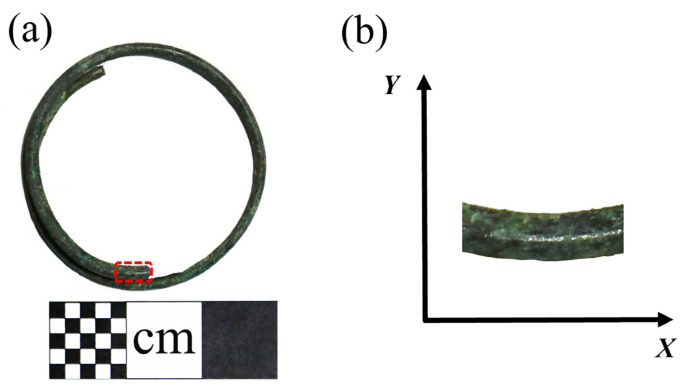
(**a**) Bronze earring sampling and (**b**) coordinate system.

This earring, crafted from copper wire wound into a circular plane with an overlapped joint, has an external diameter of 3.18 cm. The cross-section of the copper wire is almost circular, with a diameter of 0.18 cm. The bronze earrings are shown in Figure 2a and the sampling and coordinate system are shown in Figure 2b.

### 2.2. Methods

Samples were sectioned along the longitudinal cross-section (Figure 2) and sequentially polished with metallographic sandpapers of 800#, 1200#, 2000#, and 4000# for rough and fine polishing. Subsequently, the samples were polished with Al_2_O_3_ particles of 2.5, 1.5, and 0.5 μm until no scratches were visible on the surface. The samples were then corroded using a solution prepared from 1 g FeCl_3_, 3 mL HCl, and 12 mL C_2_H_6_O. Microstructures were observed using a metallographic microscope (ZEIZZ Observer A1m) and a thermal field emission scanning electron microscope (ZEISS Gemini SEM 300) equipped with an EDS detector from Oxford Instruments X-Max (Oxfordshire, UK). The analysis was conducted using Aztec 5.1 software from Oxford EDS, and a built-in virtual standard database was used. The analysis results were quantitative analysis without standard samples. The EBSD detector used was from Oxford Instruments Symmetry. The EBSD sample preparation process was based on the preparation of metallographic samples, followed by ion beam polishing. The data analysis software used was AZtecCrystal 2.1 from Oxford Instruments. Phase analysis of the samples was conducted with a PANalytical Empyrean X-ray diffractometer under conditions of Cu-Kα radiation, 40 kV voltage, 40 mA current, a scanning speed of 5°/min, and a step size of 0.02°. Electrochemical measurements were performed with a CHI660E workstation from Shanghai Chenhua, Shanghai, China; NaCl (Tianjin Fuchen Chemical Reagents, Ltd., Tianjin, China); AgCl electrode (Shanghai Xianren Chemical Reagents, Ltd., Shanghai, China); platinum electrode (PE, homemade); and deionized water (homemade). Electrochemical impedance spectroscopy and polarization curves were utilized to analyze the effects of the different soaking times and chloride ion concentrations on the corrosion of bronze alloys. Electrochemical impedance spectroscopy tests were conducted at a stable open circuit potential, with frequencies ranging from 100 kHz to 0.1 Hz and a disturbance signal amplitude of 10 mV. Polarization curves were scanned at a rate of 20 mV/min, testing within a range of ±400 mV around the open circuit potential.

## 3. Results and Discussion

### 3.1. Structure and Composition

**Figure 3:**
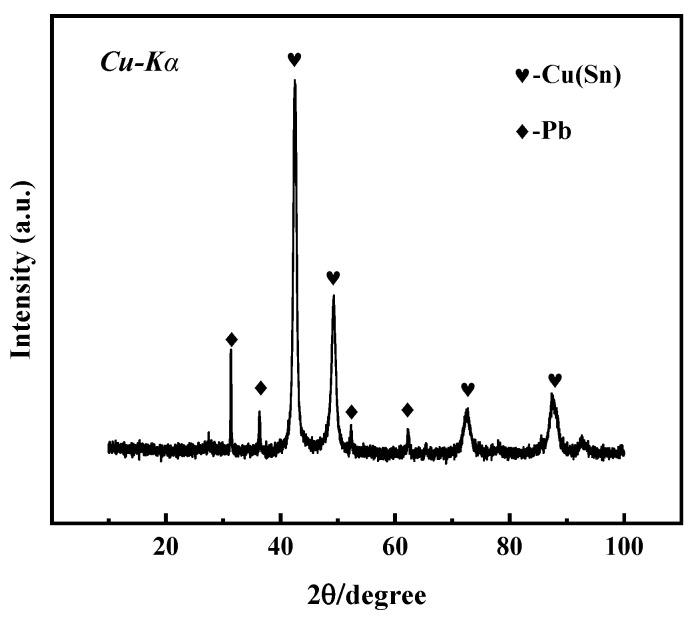
XRD pattern of bronze earrings.

The microstructure, structure, and properties of the samples are closely related to their composition. Figure 3 shows the X-ray diffraction spectrum of the bronze earrings. Comprehensive analysis of the X-ray diffraction spectra and energy spectrum results of the alloy samples indicates that the samples are predominantly composed of face-centered cubic structured α-Cu(Sn) and Pb phases. This result not only reveals the microstructural characteristics of the samples but also provides an important material basis for further understanding the corrosion behavior of bronze earrings.

**Figure 4:**
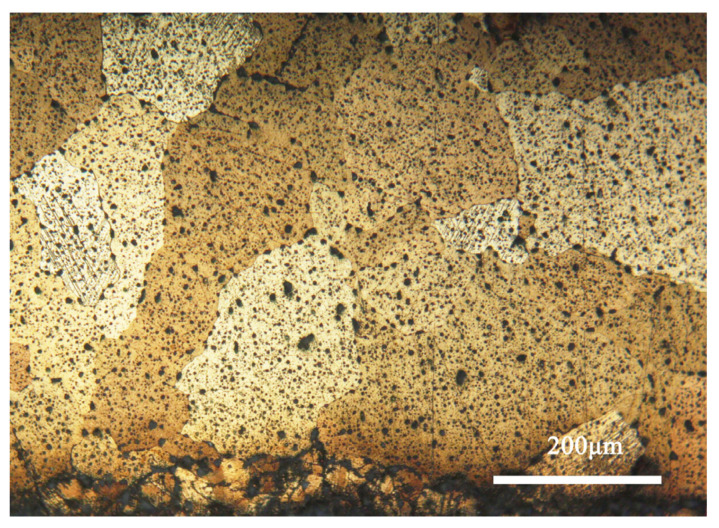
Optical micrograph image of the bronze earring.

Figure 4 shows the metallographic morphology of the bronze earring samples. It can be observed that the samples predominantly display structures formed during the casting process, with larger grains present in the central area, whereas areas closer to the surface are rich in equiaxed and twinned structures. The samples contain α-Cu(Sn) solid solution and second-phase Pb particles. Additionally, the images reveal defects and shrinkage porosity that may occur in the bronze alloys during casting, which is significant for understanding the microstructure and properties of the material [28].

To further investigate the mechanism of grain formation within the alloy, this study utilized Electron Backscatter Diffraction (EBSD) technology to reconstruct the grain structure of the bronze earrings’ X-Y section in detail.

EBSD is an efficient technique for studying the crystallographic information of alloy materials in the micro region [29]. By using EBSD technology, the orientation imaging information of the sample can be obtained, thereby obtaining richer internal information of the sample. Combined with EDS, the morphology, composition, and structure of the sample can be obtained simultaneously [30]. This article uses EBSD technology to detect and analyze bronze earrings unearthed from the Xindianzi Cemetery, studying their phase, twinning, and grain size and shape. The composition of different phases and inclusions in the alloy was obtained by combining SEM-EDS technology [31].

**Figure 5:**
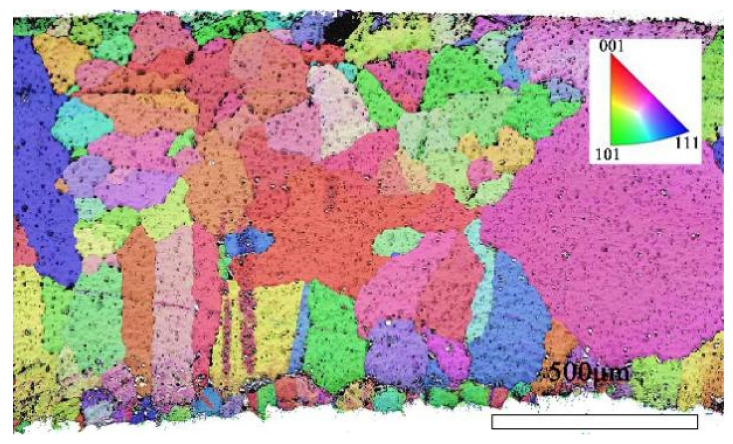
EBSD orientation map of the X-Y section of the bronze earring.

**Figure 6:**
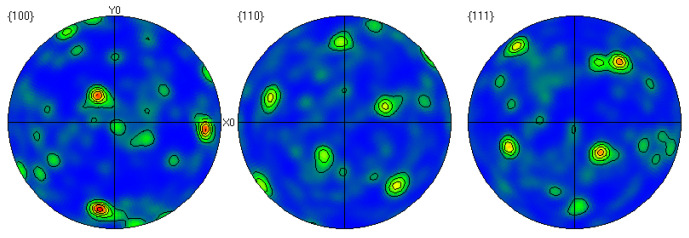
Polar diagram of the cross-section of the bronze earring.

Through EBSD image (Figure 5), we could clearly observe the grain orientation distribution along the Z direction on the X-Y section, for which the attached orientation legend and 500 μm scale provides detailed visual references. The application of EBSD data processing software (AZtecCrystal 2.1) enabled us to deeply analyze the microstructural characteristics of the bronze earrings from various aspects such as pole figures, grain size and shape, orientation difference distribution, and phase distribution. Through the analysis of the poles in particular, shown in Figure 6, we found the earrings have a specific (214)<12-1> texture, indicating the crystals exhibit a certain preferential orientation in specific directions.

**Figure 7:**
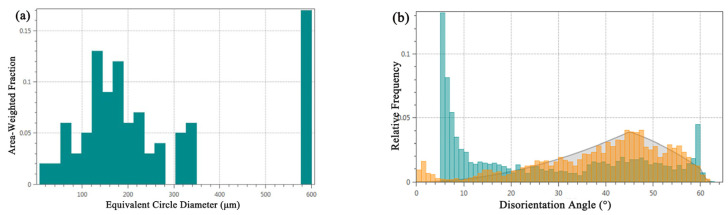
Microstructural characteristics of bronze earrings: (**a**) map of grain size analysis and (**b**) map of orientation difference distribution.

Grain size, as a key parameter for assessing material performance, is meticulously analyzed using EBSD technology, which provides extensive information on grain boundaries, enabling precise analysis of grain size and shape within the tested area. The grain size distribution, illustrated in Figure 7a, reveals an average grain size of 35.3 μm for the alloy, ranging from a minimum of 7.1 μm to a maximum of 577.9 μm. Notably, larger grains predominantly occupy the central region of the alloy, displaying significantly larger grain sizes compared to typical bronze casting grains, without any observed segregation phenomena. Conversely, the alloy’s lower surface vicinity contains a higher presence of smaller grains. According to the Hall-Petch mechanism, the material’s yield strength is inversely proportional to the square root of the grain size, suggesting that the mechanical properties of the bronze earrings significantly improves in regions closer to the lower surface.

Figure 7b shows the distribution of the grain boundary orientation differences in the tested area of the bronze earring. A noticeable increase in the number of grain boundaries is observed when the orientation difference approaches 60°, which corresponds to the twin boundary orientation difference of 60° in face-centered cubic Cu(Sn) alloys, indicating the presence of twinning structures in the alloy. Analysis of the distribution of different orientation differences using EBSD data processing software shows that low-angle boundaries (orientation difference less than 15°) account for approximately 76.6% of the total grain boundaries, whereas high-angle boundaries account for about 23.4%. Compared to the theoretical random orientation difference distribution (generally considered to be around 45°), the proportion of low-angle grain boundaries in this alloy is higher.

**Figure 8:**
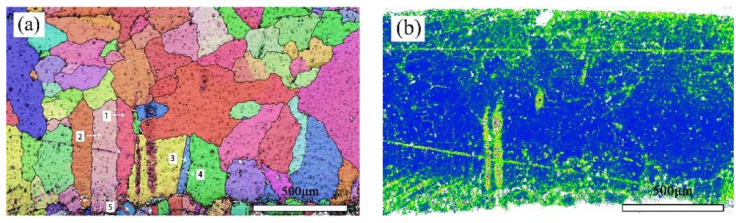
EBSD characterization of the bronze earring: (**a**) Local orientation and (**b**) KAM map.

Figure 8a shows the crystal orientation in a local area of the bronze earring, revealing the presence of equiaxed and twinned structures near the alloy surface. By measuring the orientation differences at the positions of twins, it was found that the orientation differences at the third and fifth positions of the alloy matrix are 59.28 degrees and 58.77 degrees, respectively, matching the twinning relationship of <111> crystal direction in α-Cu phase, whereas the orientation differences at other positions are less than 60 degrees. Additionally, EBSD technology was used not only for qualitative analysis of twins in the alloy but also for quantitative studies, revealing a higher twin boundary density in the lower surface area of the alloy. The introduction of KAM (kernel average misorientation) distribution maps further explored the degree of deformation in different areas inside the bronze earrings [32]. KAM distribution maps qualitatively describe the inhomogeneity of plastic deformation and the distribution of defect density, showing higher KAM values at the top and bottom surfaces of the alloy, indicating higher degrees of deformation in these areas. EBSD results (Figure 8a) indicate the presence of more twins and equiaxed crystals on the lower surface of the bronze earring, characteristic of annealing twins, suggesting that the alloy surface underwent heat treatment during its manufacturing process.

**Figure 9:**
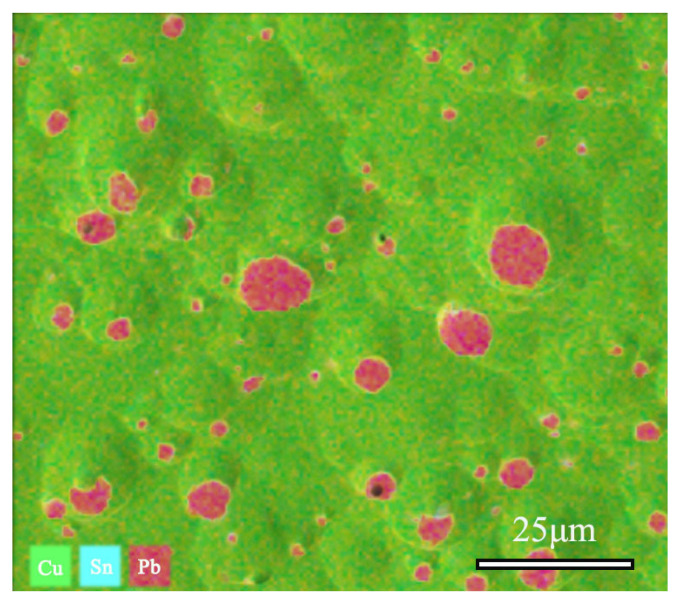
SEM-EDS and Cu, Sn, Pb elements distribution Map of bronze earrings (Cu78.36%, Sn10.71%, Pb10.82%).

SEM-EDS was utilized to collect and test the distribution maps of different elements in the area, as shown in Figure 9. Quantitative analysis revealed the composition of the sample, with Cu accounting for 78.36%, Sn at 10.71%, and Pb at 10.82%. The EDS spectrum analysis results indicate that the alloy’s matrix is a Cu-Sn solid solution constituted by Cu and Sn elements, whereas Pb elements in the alloy are distributed in the form of particles.

**Figure 10:**
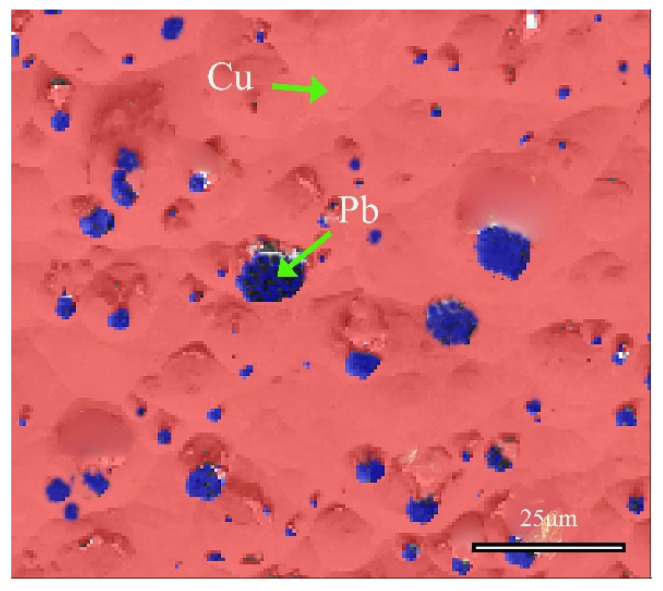
Phase distribution map of the local area of the bronze earring by EBSD.

Figure 10 shows the distribution information of local phases in the alloy, and further analysis confirmed the phase of the matrix and the second phase. The red region represents the face-centered cubic structure of α-Cu(Sn) solid solution, whereas the blue region refers to the face-centered cubic Pb element. These SEM-EDS analysis results, combined with EBSD and XRD analysis, collectively confirmed the structural information of the matrix and second phase of the bronze earrings. In the Cu-Sn-Pb ternary alloy, Pb does not form a solid solution with the Cu alloy but is distributed in a separated state, which positively affects the alloy’s solidification properties, although adding Pb to Cu-Sn alloys may affect the mechanical properties such as the strength and hardness of the bronzeware [33].

### 3.2. Corrosion Products

**Figure 11:**
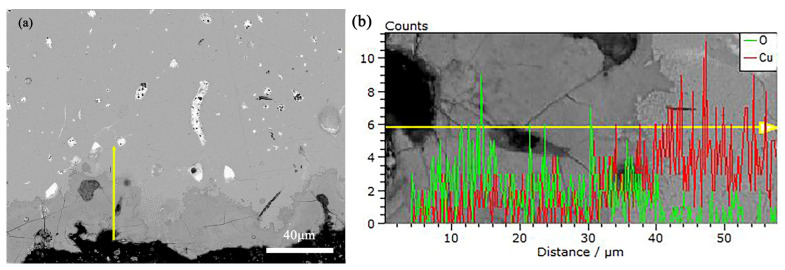
(**a**) SEM-EDS of Bronze Earrings and (**b**) the distribution of Cu and O Element Lines.

To deeply understand the corrosion process and products of alloys, this study employed Scanning Electron Microscope-Energy Dispersive Spectroscopy (SEM-EDS) to analyze the pre- and post-corrosion states of bronze earrings. Regarding the issue of whether there are signs of active corrosion, analysis was conducted on the corrosion products using SEM-EDS. Figure 11 shows the SEM-EDS image of the corrosion layer near the surface of the earring before corrosion, as well as the distribution of Cu and O element lines. Through SEM-EDS analysis of rust products, the results showed that the corrosion near the sample surface was more severe, with a thickness of 10–40 microns. The oxygen content of the rust layer was significantly higher than that of the substrate, and the copper content of the rust layer was significantly lower than that of the substrate.

**Figure 12:**
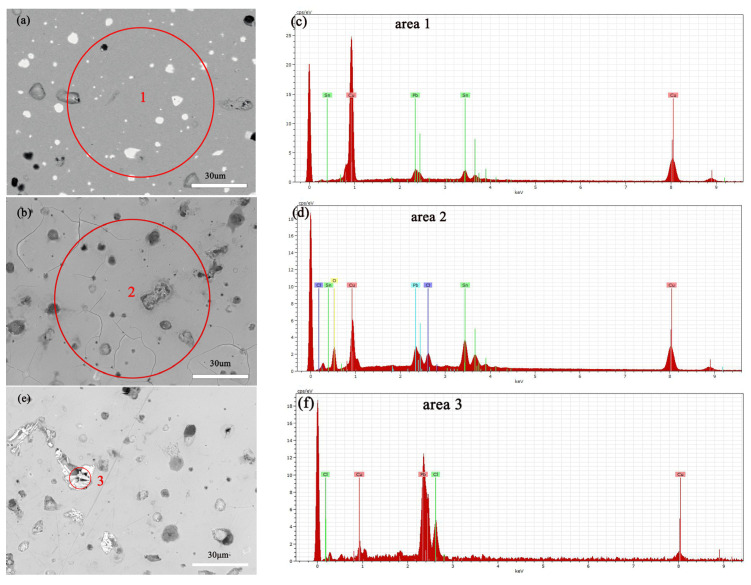
SEM-EDS images and elemental energy spectra of the bronze earrings before immersion (**a**,**c**) and after immersion for 3 days in 0.5 mol/L NaCl solution (**b**,**d**–**f**).

Figure 12a,b show the SEM images of the alloy before and after soaking in 0.5 mol/L NaCl solution for 3 days, with a significant increase in surface erosion confirmed by SEM-EDS compositional analysis, the results of which are shown in Table 1. Specifically, the copper (Cu) content decreased, whereas chloride (Cl), tin (Sn), and oxygen (O) contents increased significantly, indicating the progression of corrosion reactions. Electrochemical testing further revealed how an increase in NaCl solution concentration accelerates the dissolution of copper elements in Cu(Sn) alloys [34]. Chloride ions play a decisive role in the corrosion of copper alloys in sodium chloride solutions, with increased chloride ion concentration promoting the corrosion rate of the bronze alloy matrix. Copper elements are corroded as anodes to form CuCl [35,36], an unstable intermediate product that releases chloride ions during hydrolysis and oxidation, leading to continuous dissolution of the bronze alloy matrix [37], and forming a Cu_2_O protective layer on the alloy surface [34,38]. According to Figure 12c, it can be seen that after soaking the alloy in a 0.5 mol/L NaCl solution, deep corrosion pits appear at the Pb particles, surrounded by granular and needle like products. The EDS results indicate that the position with high lead content has a higher Cl ion content than the CuSn solid solution, the CuSn phase did not undergo significant corrosion dissolution. The needle shaped corrosion products contain three elements: Pb, O, and Cl, with the highest Pb content. After corrosion of Pb, the Pb element transfers from the original position of Pb particles, forming corrosion pits, which can accelerate the corrosion of the sample [23].

**Figure 13:**
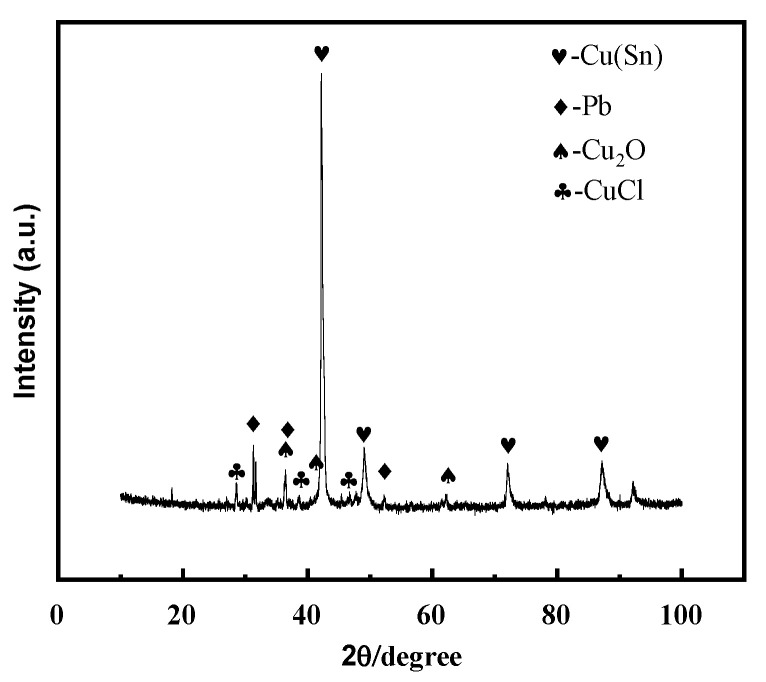
XRD pattern of copper–tin–lead alloy soaked in 0.5 mol/L NaCl solution for 3 days.

An alloy with a copper–tin–lead content similar to that of earrings was prepared by arc melting. The XRD diffraction patterns of the alloy before and after immersion in a 0.5 mol/L solution were compared, and it was found that the surface of the alloy after immersion contained CuCl and Cu_2_O phases (Figure 13).

### 3.3. Electrochemical Corrosion Characteristics

**Figure 14:**
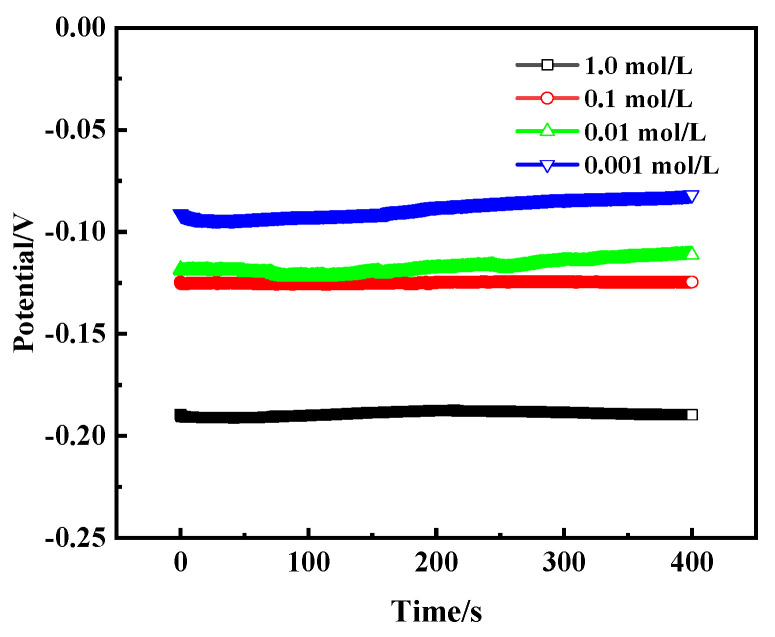
OCP curves of bronze earrings in NaCl solutions.

Figure 14 shows the open circuit potential (OCP) curves of bronze earrings in NaCl solutions of different concentrations, revealing the trend that the self-corrosion potential changes with the concentration of NaCl. As the concentration of NaCl solution increases, the self-corrosion potential of the bronze alloy also increases accordingly, specifically from −0.09 V to −0.19 V. This change illustrates the direct impact of salt concentration on the corrosion rate of bronze alloys, with high concentrations of NaCl solution accelerating the corrosion process, making the protection of the alloy more difficult.

**Figure 15:**
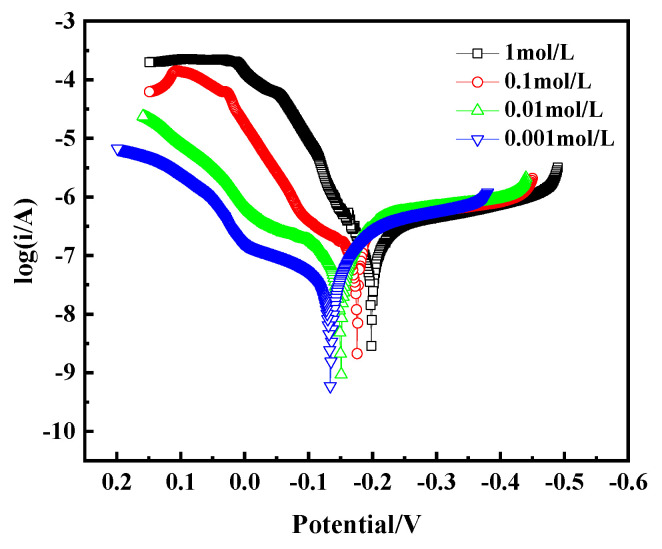
Tafel curves of bronze earrings in NaCl solutions.

The Tafel curve, as a key tool for assessing alloy corrosion behavior, is crucial for analyzing the corrosion mechanism of bronze earrings in NaCl solutions of different concentrations. By plotting the Tafel curve, as shown in Figure 15, we observe that with the increase in NaCl concentration, the corrosion current density of the bronze earrings also increases correspondingly. This phenomenon intuitively demonstrates the positive correlation between NaCl concentration and the corrosion rate of the bronze earrings. The increase in corrosion current density signifies the enhanced corrosion activity of the alloy in environments with higher NaCl concentrations.

The polarization curves were fitted using CHI660E software (CHI Version 14.05), and the results are shown in Table 2. The fitting indicates that the corrosion current density of bronze increases from 2.372 × 10^−7^ A/cm^2^ to 9.051 × 10^−7^ A/cm^2^ as the NaCl concentration increases from 10^−3^ mol/L to 1 mol/L. The polarization resistance (*Rp*) of the bronze alloy in different concentration solutions was calculated using the Stern equation, *Rp* = *Ba* × *Bc*/[2.3(*Ba* + *Bc*) *× I*_corr_] (in which *Ba* and *Bc* are the Tafel slopes of the anode and cathode, respectively, and *Rp* is the polarization resistance). The results showed that *Rp* decreases with increasing NaCl concentration, indicating that the bronze alloy exhibits better corrosion resistance in low-concentration NaCl solutions.

Figure 18a illustrates the equivalent circuit diagram for bronze alloys in NaCl solution, modeling the electrochemical kinetics of the corrosion process on the bronze surface. In this circuit, Rs represents the solution resistance of the NaCl solution, indicating its conductivity; RCT represents the charge transfer resistance, inversely related to the corrosion rate of the bronze alloy surface, meaning a higher RCT indicates better corrosion resistance; and the constant phase element (CPE), denoted as Qdl, simulates the double-layer capacitance and its non-ideal behavior between the electrolyte and bronze surface. Fitting the electrochemical parameters of bronze earrings in various NaCl concentrations (Table 3) revealed that as NaCl concentration increases, Rs decreases, reflecting increased solution conductivity; meanwhile, changes in RCT suggest the increase in chloride ion concentration promotes the dissolution of copper elements in the alloy, accelerating the corrosion process of the bronze matrix.

**Figure 16:**
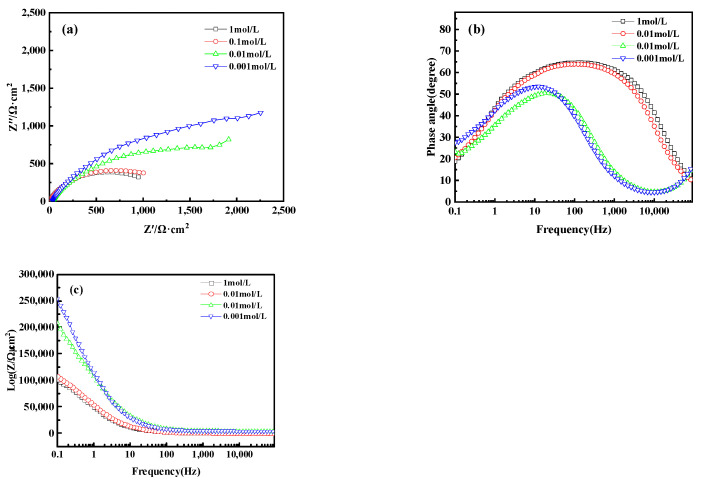
(**a**) Nyquist plot and (**b**,**c**) Bode of bronze earrings in NaCl solutions.

Electrochemical Impedance Spectroscopy (EIS) analysis further revealed the corrosion behavior of bronze earrings in various concentrations of NaCl solutions. Figure 16a presents the Nyquist plots of the electrochemical impedance spectra for the bronze earrings in different NaCl solution concentrations. The Nyquist plots demonstrate that with an increase in NaCl concentration, the capacitive loop of the alloy decreases, suggesting a reduction in charge transfer resistance and an increase in corrosion rate. Analysis of the Bode plot in Figure 16b reveals that after soaking for 1 h in NaCl concentrations of 10^−3^, 10^−2^, 10^−1^, and 1 mol/L, no oxide film formed on the surface of the bronze alloy, as indicated by the presence of a single time constant. Moreover, the Bode magnitude plot in Figure 16c indicates that the impedance values Z decrease progressively with increasing NaCl concentration, further confirming an increase in the corrosion rate.

**Figure 17:**
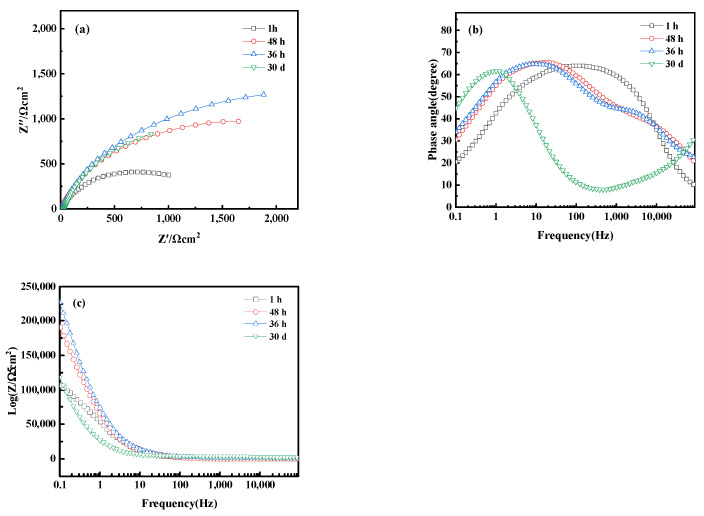
(**a**) Nyquist plot and (**b**,**c**) Bode of bronze earrings after immersion for different times in 0.5 mol/L NaCl solution.

Figure 17 shows the electrochemical Nyquist and Bode plots of bronze alloy in 0.5 mol/L NaCl solution over different immersion times, including 1 h, 48 h, 36 h, and 30 days. Within 36 h, the capacitive arc of the alloy increases with immersion time, indicating the formation of a protective oxide film during the corrosion process. However, when the immersion time extends to 30 days, the capacitive arc decreases, indicating a reduction in corrosion rate. The Bode plot shows that at the initial stage of immersion (1 h), the alloy primarily exhibits a time constant in the high-frequency region, suggesting the activation reaction of the metal matrix without oxide film formation. Increasing the immersion time to 36 h, the Bode plot reveals two time constants: the high-frequency region corresponds to the reaction at the passive film interface, whereas the mid-to-low frequency region corresponds to the corrosion process of the metal matrix, indicating that the formation of the oxide film to some extent inhibits corrosion. As immersion time increases, the impedance magnitude of the alloy first increases and then decreases, reflecting the dynamic changes in the corrosion process. These results reveal the time-dependent corrosion behavior of bronze alloys in sodium chloride solutions and the formation and stability of protective oxide films. In addition, chlorides are a significant reason for the corrosion of iron cultural relics and the instability of iron cultural relics. Determining the types and properties of chlorides, and then selecting effective methods for control, dechlorination, or conversion treatment, is crucial in the protection process of iron cultural relics [39]. These studies contribute to understanding the long-term corrosion mechanisms of bronze alloys.

**Figure 18:**
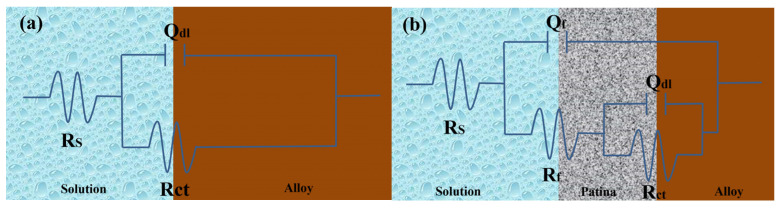
Equivalent circuits show the bronze earrings soaked in 0.5 mol/L NaCl initially (**a**) and for 3 days (**b**) [34].

From the Electrochemical Impedance Spectroscopy (EIS) tests and their fitting results conducted in NaCl solutions of different concentrations, it was observed that with an increase in chloride ion concentration in the NaCl solution, the charge transfer resistance (RCT) significantly decreases, indicating that the rise in chloride ion concentration notably accelerates the dissolution process of copper. The consistency among Open Circuit Potential (OCP), Tafel curves, and EIS test results further confirms the role of increased chloride ion concentration in promoting the corrosion rate of the bronze matrix. Particularly, in a 0.5 mol/L NaCl solution, the corrosion behavior of bronze was initially uniform, but after 3 days, a protective film formed on the alloy surface. By comprehensively analyzing these experimental results, we can detail the corrosion behavior of bronze in NaCl solution over time and thus draw the corresponding equivalent circuit diagram, as shown in Figure 18. The experimental outcomes indicate that at the initial stage of immersion in NaCl solution, a protective film has not yet formed on the alloy surface, but as the immersion time extends, a protective film gradually forms. Chloride ions play a decisive role in the corrosion mechanism of copper alloys, not only accelerating the corrosion process of bronze alloys but also significantly affecting the rate and morphology of corrosion through activating the reactivity of the bronze alloy surface. This process involves the facilitation of electrochemical reactions, in which chloride ions act as catalysts, promoting the formation of copper oxides and chlorides on the alloy surface [40,41]. Therefore, the prevention of bronze corrosion should focus on the stability of CuCl and the removal of chloride ions.

## 4. Conclusions

This study thoroughly analyzes the structural characteristics of the bronze earrings unearthed from Tomb M47 at Xindianzi, as well as their electrochemical corrosion behavior in NaCl solutions of various concentrations. The comprehensive analysis of the bronze earrings revealed that their matrix is mainly composed of face-centered cubic Cu(Sn) solid solution with a certain amount of Pb particles. EBSD techniques showed that the earrings’ grains exhibit specific orientations, namely, the (214)<12-1> texture, with more twins and equiaxed grains at the lower surface position, indicating annealing twins. Furthermore, this indicates that the bronze alloy undergoes thermal processing, resulting in specific (214)<12-1>textures on the earrings. Moreover, this suggests that the samples underwent thermal treatment during their manufacturing process, affecting their microstructure and corrosion behavior. Electrochemical testing results indicated that the corrosion rate of the bronze alloy significantly increases with the NaCl solution concentration from 10^−3^ to 1 mol/L, highlighting the key role of chloride ions in promoting the corrosion process of bronze alloys. Notably, immersion experiments in 0.5% NaCl solution showed that the loss of Cu in the alloy became more severe after 3 days of immersion, with the alloy surface forming an oxide film containing CuCl and Cu_2_O. Research has shown that reducing the conversion of CuCl and removing chloride ions is an important factor for improving the stability of bronze. These results provide important scientific evidence for revealing the corrosion mechanism of bronze earrings in different environments.

## Figures and Tables

**Table 1:** SEM-EDS composition analysis results of the bronze earrings before immersion (a) and after immersion for 3 days in 0.5 mol/L NaCl solution (b).

Position	Sn (wt.%)	Cu (wt.%)	Pb (wt.%)	O (wt.%)	Cl (wt.%)
Without corrosion	10.71	78.36	10.82	0.11	0
With corrosion	20.60	55.16	11.14	9.73	2.76

**Table 2:** Fitting parameters of polarization curves for bronze earrings in NaCl solutions.

Solution	*E*_corr_/V	*I*_corr_/A·cm^2^	*Ba*/V^−1^	*Bc*/V^−1^	*Rp*/KΩ
1 mol/L	−0.196	9.051 × 10^−7^	2.155	16.3	91.432
10^−1^ mol/L	−0.172	6.204 × 10^−7^	3.353	5.816	149.044
10^−2^ mol/L	−0.145	5.795 × 10^−7^	9.626	3.948	210.041
10^−3^ mol/L	−0.130	2.372 × 10^−7^	10.519	3.776	509.386

**Table 3:** EIS fitting results of bronze earrings in NaCl solutions.

Solution	Rs/(Ω·cm^2^)	Qdl/(Ω·cm^2^)	n	Rct (Ω·cm^2^)
1 mol/L	47.200	5.0805 × 10^−8^	0.742	115,590
10^−1^ mol/L	71.570	3.5577 × 10^−6^	0.737	123,000
10^−2^ mol/L	3347	1.8811 × 10^−6^	0.687	230,200
10^−3^ mol/L	3454	1.9862 × 10^−6^	0.705	319,460

## Data Availability

The data that support the findings of this study are available from the corresponding author upon request (due to privacy).
